# Shock index as predictor of massive transfusion and mortality in patients with trauma: a systematic review and meta-analysis

**DOI:** 10.1186/s13054-023-04386-w

**Published:** 2023-03-05

**Authors:** Andrea Carsetti, Riccardo Antolini, Erika Casarotta, Elisa Damiani, Francesco Gasparri, Benedetto Marini, Erica Adrario, Abele Donati

**Affiliations:** 1 Department of Biomedical Sciences and Public Health, Università Politecnica delle Marche, Ancona, Italy; 2 Anesthesia and Intensive Care Unit, Azienda Ospedaliero Universitaria delle Marche, Ancona, Italy

**Keywords:** Shock index, Massive transfusion, Mortality, Hemorrhagic shock, Trauma

## Abstract

**Background:**

Management of bleeding trauma patients is still a difficult challenge. Massive transfusion (MT) requires resources to ensure the safety and timely delivery of blood products. Early prediction of MT need may be useful to shorten the time process of blood product preparation. The primary aim of this study was to assess the accuracy of shock index to predict the need for MT in adult patients with trauma. For the same population, we also assessed the accuracy of SI to predict mortality.

**Methods:**

This systematic review and meta-analysis was performed in accordance with the PRISMA guidelines. We performed a systematic search on MEDLINE, Scopus, and Web of Science from inception to March 2022. Studies were included if they reported MT or mortality with SI recorded at arrival in the field or the emergency department. The risk of bias was assessed using the QUADAS-2.

**Results:**

Thirty-five studies were included in the systematic review and meta-analysis, for a total of 670,728 patients. For MT the overall sensibility was 0.68 [0.57; 0.76], the overall specificity was 0.84 [0.79; 0.88] and the AUC was 0.85 [0.81; 0.88]. Positive and Negative Likelihood Ratio (LR+; LR−) were 4.24 [3.18–5.65] and 0.39 [0.29–0.52], respectively. For mortality the overall sensibility was 0.358 [0.238; 0.498] the overall specificity 0.742 [0.656; 0.813] and the AUC 0.553 (confidence region for sensitivity given specificity: [0.4014; 0.6759]; confidence region for specificity given sensitivity: [0.4799; 0.6332]). LR+ and LR− were 1.39 [1.36–1.42] and 0.87 [0.85–0.89], respectively.

**Conclusions:**

Our study demonstrated that SI may have a limited role as the sole tool to predict the need for MT in adult trauma patients. SI is not accurate to predict mortality but may have a role to identify patients with a low risk of mortality.

**Supplementary Information:**

The online version contains supplementary material available at 10.1186/s13054-023-04386-w.

## Background

Injuries are responsible for about 8% of all deaths worldwide [1]. Hemorrhage is often preventable death and early hemorrhage control pathway including time-critical transfusion is crucial to reduce this preventable mortality. The early recognition and management of bleeding patients is the major challenge because rapid intervention aiming to stop the bleeding is fundamental to reducing mortality and morbidity. Prediction of the need for a hemorrhage control pathway should be easy, quick and reliable to anticipate and mobilize resources. Massive transfusion (MT) requires resources to ensure the safety and timely delivery of blood products [2]. Early prediction of MT is useful to anticipate the process of blood product preparation [2]. Thus, MT protocols improve patients’ outcome and minimized blood product wastage [3, 4]. Several scores to predict MT have been proposed [5], but they may not be always simple to be calculated in the field. The Shock Index (SI) is a simple mathematical equation based on the ratio between heart rate (HR) and systolic blood pressure (SBP), it is easy to calculate in pre-hospital settings and those vital signs are frequently communicated to the trauma center directly from the scene. Normal values in adults range from 0.5 to 0.7. A hypovolemic shock classification based on this score has been proposed (SI < 0.6: no shock; SI 0.6–1: mild shock; SI 1–1.4: moderate shock; SI ≥ 1.4: severe shock) [6]. The current European guidelines on the management of major bleeding and coagulopathy following trauma suggest using SI to assess the severity of hypovolemic shock [7].

We performed a systematic review and meta-analysis to investigate the role of SI to predict the need for MT in adult trauma patients. The secondary aim was to assess the role of SI to predict mortality in patients with trauma.

## Methods

### Protocol and guidance for conducting and reporting

The Preferred Reporting Items for Systematic Review and Meta-Analysis Protocols (PRISMA-P) guidelines [8] have been followed to write the protocol while the methodology for conducting and reporting the systematic review was based on the Preferred Reporting Items for Systematic Reviews and Meta-analyses of diagnostic test accuracy studies (PRISMA-DTA) guidelines [9]. The protocol (Additional file 1) has been registered on International Prospective Register of Systematic Reviews (PROSPERO) (CRD42021270285).

### Review questions and hypothesis

The primary review question was if SI could predict the need for MT in adult patients with trauma, while the secondary review question was if SI could predict the mortality of adult patients with trauma.

We hypothesized that SI was significantly higher in adult trauma patients that need MT and in those that did not survive to trauma.

### Primary and secondary outcomes

The primary outcome of the study was to assess the accuracy of SI to detect the need for MT in adult patients with trauma.

The secondary outcome was to assess the accuracy of SI to detect mortality in adult patients with trauma.

### Eligibility criteria

We considered patients with trauma, and ≥ 14 years old. No further exclusion criteria different from pediatric age (< 14 years) have been considered. We selected papers that reported pre-hospital and/or hospital SI and the need for MT. MT is classically defined as a transfusion of > 10 red blood cells (RBC) units in 24 h. However, because of MT definition is evolving [10], we did not restrict it to a specific definition, but we considered MT as defined by the single study. For the secondary outcome, mortality (as defined in the study) was recorded when available.

Randomized controlled trials and observational studies (prospective and retrospective) were included. Conference proceedings, abstracts, case reports, and studies not involving humans were excluded. No language restrictions were applied.

Studies with data from the field and the hospital are included.

### Search strategy

We searched the Medline, Scopus, and Web of Science databases from their inception to September 2022. The search strategy can be found in the Additional file 1. Reference lists of eligible studies and review articles have been assessed. Before paper submission, the research was repeated (1 December 2022) to identify further papers published in the meantime, and the analysis was updated.

### Study selection and data extraction

Two researchers (A.C. and R.A.) independently screened titles and abstracts of all papers resulting from the database search. Subsequently, they independently assessed the full text of the papers selected from titles and abstracts screening. The same investigators independently performed data extraction. Any discrepancies that arose during the selection process and data extraction were solved by consensus or by the decision of a third independent researcher (A.D.). CADIMA software vers. 2.2.3 (JKI—Julius Kühn-Institut) has been used for screening and papers assessment.

### Assessment of risk of bias and quality of the evidence

Two trained investigators (A.C. and A.R.) independently rated the quality of the selected studies. As per the Cochrane DTA handbook [11], the quality assessment of diagnostic accuracy studies (QUADAS-2) tool was used to assess for risk of bias and applicability concerns in patients’ selection, index test, reference standard, and flow and timing. Each item was evaluated as low, unclear, or high risk of bias [12]. The highest risk of bias shown for any item was used to determine the overall risk of bias for the study. The overall quality of the evidence for the primary and secondary outcomes has been assessed according to Grading of Recommendations Assessment, Development and Evaluation (GRADE) guidelines [13, 14].

### Statistical analysis

The statistical analysis has been performed using diagmeta package in R (R version 4.2.0 (2022-04-22)), and metandi and midas in STATA (StataCorp 2021; Stata Statistical Software: Release 17; StataCorp LLC). The bivariate model proposed by Reitsma et al. has been used to assess the accuracy of SI to predict MT and mortality and for summary receiver operating characteristic (SROC) curve calculation [15]. When appropriate, SROC will be drawn with confidence and prediction contour. They are both graphical representations that describe the uncertainty in the SROC estimate. The confidence contour describes the uncertainty in the SROC estimate and shows the 95% confidence interval for the SROC at a given point. The prediction contour, on the other hand, describes the uncertainty in predicting the SROC for future tests that might be performed. It shows a 95% prediction interval for the SROC, based on the distribution of SROCs estimated from different tests. This is useful for predicting future test performance and assessing the effect of any future tests. In case of multiple cut-offs for SI, the model of multiple thresholds proposed by Steinhuser has been used if appropriate [16]. This model is able to calculate the optimal cut-off.

Quantitative SROC analysis has been performed if five or more studies reported data for the primary/secondary outcome. In the presence of an appropriate number of studies, subgroup analysis considering pre-hospital SI (PH-SI) and SI recorded at hospital admission (H-SI) has been performed. Further subgroup analysis has been considered to investigate the potential sources of heterogeneity.

## Results

### Study selection and study characteristics

**Fig. 1:**
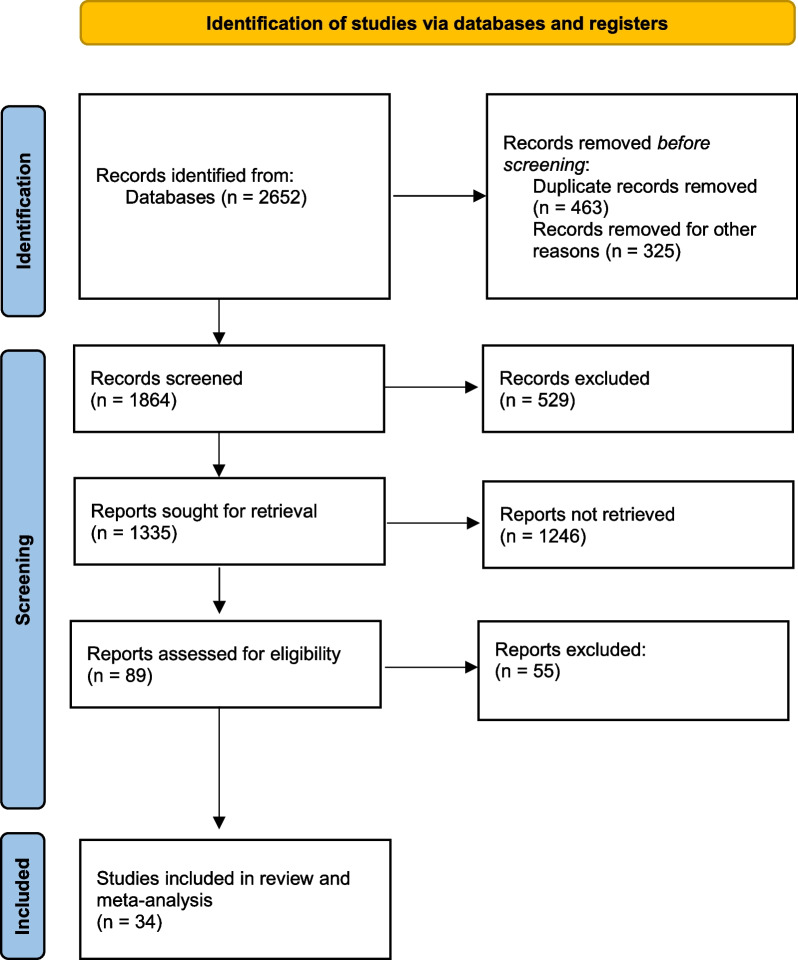
PRISMA flow diagram

2652 titles were retrieved from the literature search. After removing duplicates (*n* = 463) and papers that focused on non-trauma patients (*n* = 325), we screened the titles/abstracts of 1864 records. 529 papers have been excluded because they examined outcomes other than MT and/or mortality. Of the remaining 1335 papers, 1246 considered different tests (such as Age Shock Index and Reverse Shock Index) and were also excluded. After assessing 89 studies for eligibility, we were unable to retrieve 54 of them because they focused on a pediatric population. Finally, 35 studies were included, considering 670,728 patients (Fig. 1). Studies were published from 1996 to 2022. The main characteristics of the selected studies are described in Additional file 1: Tables S1 and S2.

All studies considered patients ≥ 14 years old involved in trauma. Twenty-two studies considered H-SI [6, 17–37] while 10 studies considered PH-SI [38–47]. Three studies reported both PH- and H-SI [48–50]. Two studies involved the geriatric population [33, 35], while Montoya et al. [36] considered only patients younger than 50 years old. Seven studies excluded patients with traumatic brain injuries (TBI) [17, 21, 32, 33, 35, 37, 41]. Some variability was observed regarding the definition of MT and mortality. Almost all studies defined MT as 10 red blood cells (RBC units within 24 h [19, 21, 22, 28, 38, 40, 42, 44, 47, 51, 52] and mortality as in-hospital mortality [18, 22, 25–29, 31, 33–35, 38, 41]. Different definitions are a further source of potential heterogeneity.

### Risk of bias and quality of evidence

**Fig. 2:**
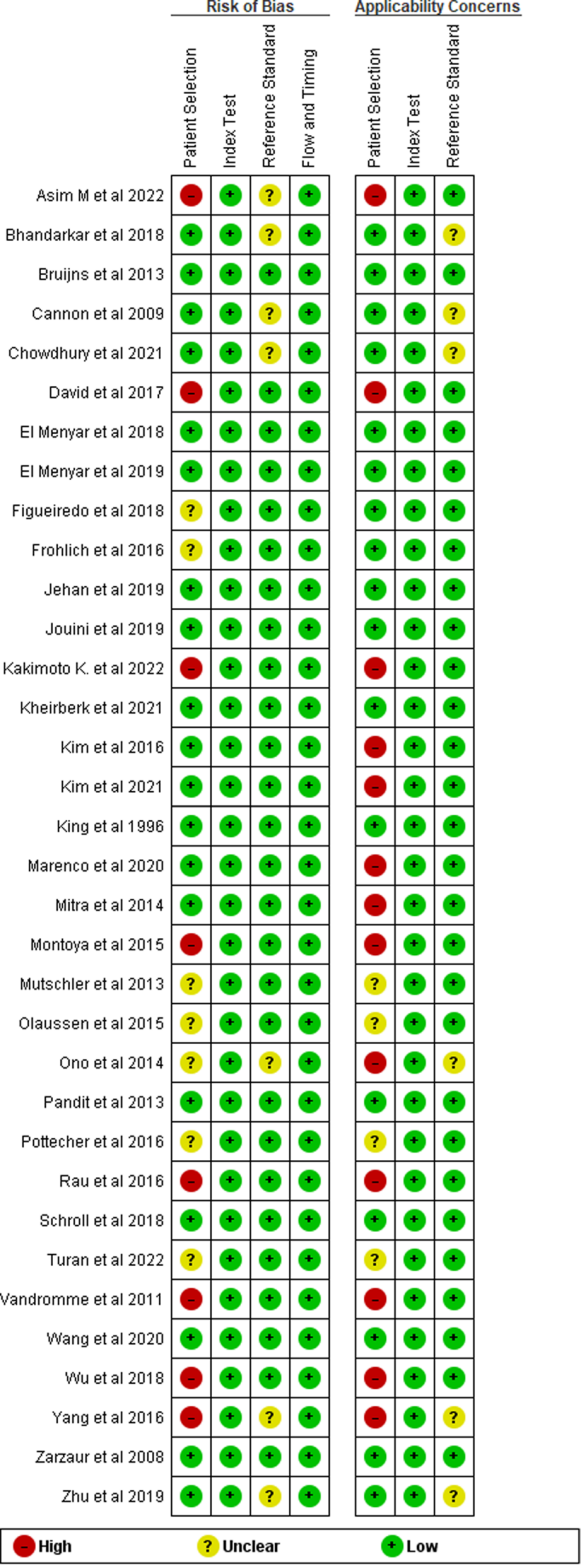
Assessment of risk of bias according to QUADAS-2

**Fig. 3:**
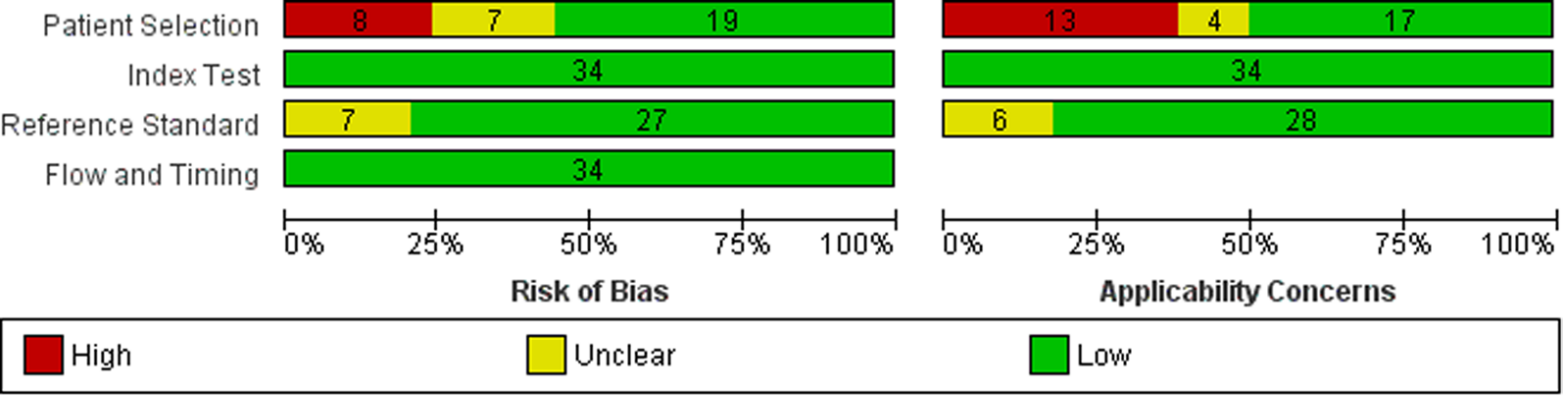
Percentage of studies for bias risk categories

The quality assessment of the studies is summarized in Figs. 2 and 3. Almost all studies clearly stated the age of the population. The overall quality of evidence was very low/low mainly due to the high heterogeneity between studies.

### Primary outcome

Fifteen studies have been considered for quantitative analysis of SROC [18, 19, 21, 22, 28, 38, 40–42, 44–48, 50].

**Fig. 4:**
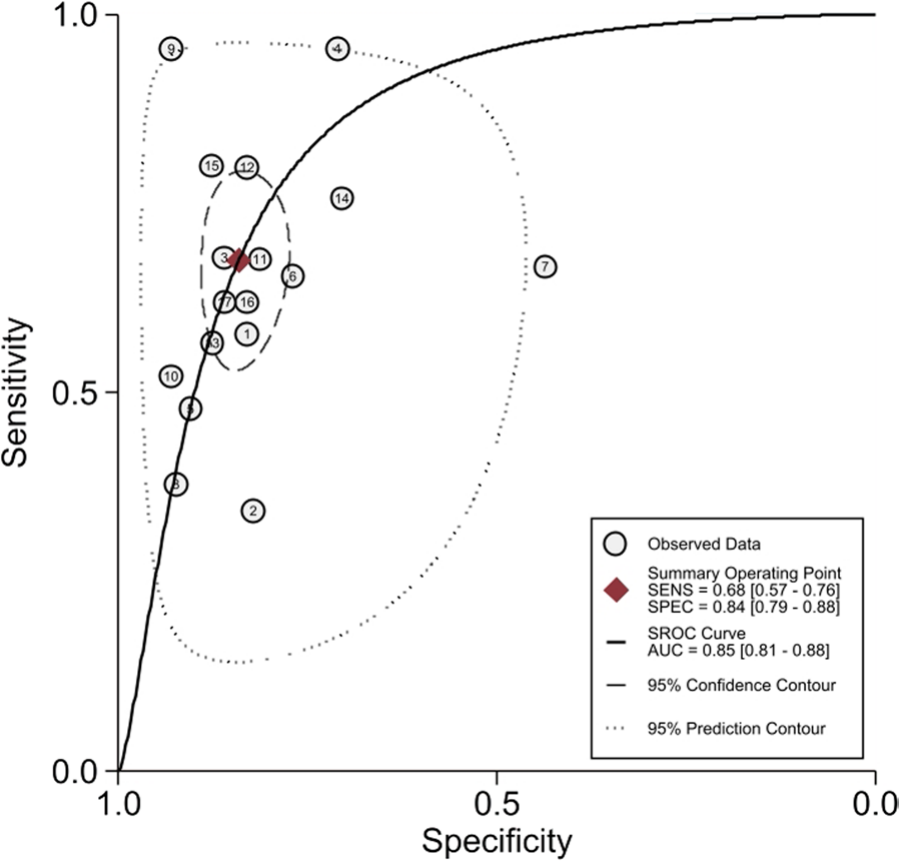
SROC for SI to predict MT

SI showed an overall sensibility of 0.68 [0.57; 0.76] and an overall specificity of 0.84 [0.79; 0.88] to predict MT. The AUC was 0.85 [0.81; 0.88] (Fig. 4). Positive and Negative Likelihood Ratio (LR+ ; LR−) were 4.24 [3.18–5.65] and 0.39 [0.29–0.52], respectively.

Considering PH-SI, the sensitivity was 0.67 [0.50; 0.81], the specificity was 0.83 [0.75; 0.89] and the AUC was 0.84 [0.81; 0.87] (Additional file 1: Figure S1). LR+ and LR− were 3.97 [2.52–6.25] and 0.40 [0.24–0.64], respectively.

Considering H-SI, the sensitivity was 0.771 [0.584; 0.890], the specificity was 0.775 [0.674; 0.852], and the AUC was 0.841 (confidence region for sensitivity given specificity: [0.4566; 0.9393]; confidence region for specificity given sensitivity: [0.7762; 0.9036]) (Additional file 1: Figure S2). LR+ and LR− were 3.43 [3.07–3.83] and 0.30 [0.27–0.34], respectively. We found an optimal cut-off value of 0.796 at hospital admission to discriminate between patients with low vs. high risk of MT.

The quality of the evidence table for SI to predict MT in adult patients with trauma is shown in Additional file 1: Table S3.

### Secondary outcome

Twenty-six studies have been considered for secondary outcome [6, 17–20, 23, 24, 26–43, 49].

**Fig. 5:**
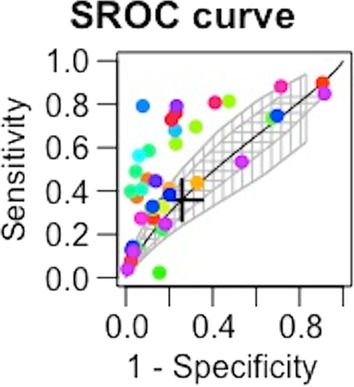
SROC for SI to predict mortality

SI showed an overall sensibility of 0.358 [0.238; 0.498] and an overall specificity of 0.742 [0.656; 0.813] to predict mortality. The AUC was 0.553 (confidence region for sensitivity given specificity: [0.4014; 0.6759]; confidence region for specificity given sensitivity: [0.4799; 0.6332]) (Fig. 5). LR+ and LR− were 1.39 [1.36–1.42] and 0.87 [0.85–0.89], respectively. The optimal cut-off of SI to predict mortality was 0.804.

Considering pre-hospital data, the overall sensibility was 0.886 [0.064; 0.998] and the overall specificity was 0.389 [0.072; 0.837]. The AUC was 0.590 (confidence region for sensitivity given specificity: [0.0632; 0.7517]; confidence region for specificity given sensitivity: [0.3237; 0.8588]) (Additional file 1: Figure S3). LR+ and LR− were 1.45 [1.38–1.52] and 0.29 [0.28–0.3], respectively. The optimal cut-off of PH-SI to predict mortality was 0.569.

Considering hospital data, the overall sensitivity was 0.462 [0.349; 0.580] and the overall specificity was 0.780 [0.699; 0.855]. The AUC was 0.638 (confidence region for sensitivity given specificity: [0.5220; 0.7333]; confidence region for specificity given sensitivity: [0.5741; 0.7037]) (Additional file 1: Figure S4). LR+ and LR− were 2.1 [2–2.18] and 0.69 [0.66–0.72], respectively. The optimal cut-off of H-SI to predict mortality was 0.836.

To reduce the potential source of heterogeneity due to different timepoint for mortality definition, we performed a subgroup analysis considering 12 studies reporting hospital mortality [18, 26–29, 31, 33–35, 38, 41, 42], as this was the most frequently reported timepoint for mortality assessment. For an optimal cut-off of 0.795, SI showed a sensitivity of 0.325 [0.161; 0.547] and a specificity of 0.736 [0.600; 0.838] to predict hospital mortality. The AUC was 0.5315 (confidence region for sensitivity given specificity [0.2712; 0.7154]; confidence region for specificity given sensitivity: [0.4157; 0.6660]) (Additional file 1: Figure S5). LR+ and LR− were 1.23 [1.19–1.27] and 0.92 [0.89–0.95], respectively.

The quality of the evidence table for SI to predict mortality in adult patients with trauma is shown in Additional file 1: Table S4.

## Discussion

Even if deaths following trauma and MT need are decreasing in the last years [53], management of massive hemorrhage is still a challenge in trauma patients. Transfusion protocols drawn up to overcome this problem improved survival [54, 55]. SI is probably one of the most useful indices that measure the severity of hypovolemia, especially when patients still present with systolic pressure in the normal range despite having suffered significant blood loss [56].

Our study showed that SI may have a role in trauma patients. It showed a high AUC of SROC curve for MT prediction. However, LR+ and LR− showed a weak ability of SI to detect whether patients needed MT or not, as a positive or negative SI reveal about 25% increased or reduced risk of MT, respectively. Both pre-hospital and hospital recordings may have approximately the same role. AUC is approximately the same for PH- and H-SI, with the PH-SI being less sensitive and more specific than H-SI. However, considering the confidence interval, this difference does not seem significant. The cut-off of 0.8 may be considered reasonable to discriminate a risk for MT.

SI values can vary in a range from 0 to infinity and increasing its value higher is the risk for transfusion. The values are therefore correlated with the degree of shock and impaired tissue perfusion [37, 57]. For this reason, SI is a better indicator of hemodynamic instability than HR or blood pressure considered individually [56]. Furthermore, an increase in SI indicates a higher probability of negative outcome and the need for more resources for treatment [49] such as surgery, mechanical ventilation, prolonged hospitalization in intensive care, and a longer hospital stay [52].

Olaussen et al. [2] support the usefulness of measuring the SI before arrival at the hospital because it could warn the physician, avoiding the preparation of unnecessary blood. According to Vandromme et al. [38], a patient who has a SI = 0.91 in the field has more than 1.5 times increased risk for receiving MT compared to a patient with normal SI, and for a SI = 1.14 in the pre-hospital setting the risk of MT is 5 times higher.

In addition to the SI, several scores would equally allow making these considerations, but the SI is the most easily accessible and calculable since it is obtained from only two parameters [38, 48]. Schroll et al. [21] have shown that SI > 1 has a greater sensitivity than the ABC score in predicting the need for MT even if it has a weaker specificity. Both Demuro et al. [58] and El-Menyar et al. [28] argue that SI > 0.8 is the better cut-off to predict the need for MT. The rationale for this choice is that it decreases the possibility of underestimating patients who may need urgent intervention during triage. However, cut-off = 1 is more specific and can be easily interpreted by pre-hospital staff since it is sufficient to consider the SBP numerically lower than the HR as a warning condition [2]. This facilitates the identification of high-risk patients without requiring other additional tools and SI > 1 could be useful criteria to suspect hemorrhagic shock [46, 48, 59–61]. The early activation of the MT protocol and its strict adherence has been shown to improve the outcome in the most serious patients and decrease the number of plasma transfusions [3]. A delay in the activation of the transfusion protocol (> 15 min from the patient's arrival) has been found in 50% of cases and it was the only cause of non-compliance with the protocol [62]. MT protocol activation based on information coming from the field could decrease delays and improve compliance.

The predictive strength of SI could be compromised in patients with chronic hypertension, diabetes mellitus, or coronary heart disease, conditions in which the dynamic response of pressure and HR—and therefore of SI—could differ from healthy patients and thus hinder its application in predicting MT [22]. Hypertension alters basal systolic blood pressure and drugs, such as beta-blockers or calcium channel blockers, limit tachycardia in response to hypovolemia [63]. Heart failure can also limit the physiological response to shock. Some authors suggest that both vital signs and SI are more difficult to interpret in the elderly because they tend to have a lower sympathetic system response in regulating HR and blood pressure. This leads to an increase in the false negative rate [32, 64, 65]. Thus, the usefulness of the SI may be limited in these subpopulations as they do not have substantial changes in HR in response to hemodynamic stress [66]. TBI has also been associated with an alteration in the autonomic response to bleeding with consequent uncoupling of the autonomic and cardiovascular systems [67, 68]. However, even if the accuracy of SI has been previously questioned by an experimental model of TBI and hemorrhage [69], a retrospective study based on a large database has confirmed the validity of SI to identify patients at risk of transfusion independently of the presence of an associated TBI [31].

Regarding the predictive capacity of SI on mortality, the selected studies have shown a low sensitivity and a higher specificity, indicating that a normal SI may be useful to identify patients with a low risk of mortality. On the other hand, an abnormal value may have limited significance. Bruijns et al. [32] analyzed the variation of some scores (SI, Age-SI, minpulse [MP], pulse max index [PMI], and blood pressure-age index [BPAI]) with mortality and they were able to show an association. Some scores consider the patient's age as a variable for calculation. Whatever the exact age, there is a broad agreement that the older the patient, the higher the probability of death, even if the patient has less severe injuries [70, 71]. A retrospective study considered 111,019 patients on beta blockers or calcium channel blockers, hypertension, diabetes and aged > 65 years to understand if these comorbidities weaken the ability of SI to predict mortality [72]. The authors have shown that patients > 65 years with SI > 1 had an increased risk of mortality at 30 days but elderly age, hypertension, and the use of beta-blockers or calcium antagonists weaken the correlation between SI and mortality [72]. Kim et al. [35] considering only an elderly population (age > 65 years), underline the utility of the Age-SI (SI multiplied by the age) to have a more precise relationship with mortality. In this case, values greater than 50 are indicative of hemodynamic instability. Pandit et al. [33] agree on the greater accuracy of the Age-SI but point out the greater difficulty in calculating this score which would make it less applicable in emergencies. Similarly, the Shock Index Pediatric Adjusted (SIPA) has been developed for the pediatric population and is more reliable for this subgroup of patients [73, 74]. The SI is the only score among those considered by Bruijns et al. [32] which has a relationship with mortality (sensitivity 0.37, specificity 0.95) without including age in its equation. According to El-Menyar et al. [28], as the SI values increase, regardless of the patient's condition, the prognosis will be worse. Higher values reflect a higher incidence of multi-organ dysfunction and sepsis, all factors affecting mortality [6, 28]. Moreover, an increase of SI > 0.3 between the pre-hospital and in-hospital recording is associated with increased mortality by 5 times [49]. Thus, considering the limitations of SI to predict mortality shown by our results, other scores should be considered. Trauma and injury severity score (TRISS) and the Revised Injury Severity Classification II (RISC II) are two more sophisticated tools specially designed for this purpose.

Recently, Need For Trauma Intervention (NFTI) has been proposed as an alternative measure to Injury Severity Score (ISS) and Revised Trauma Score (RTS) to better define major trauma [75, 76]. The NFTI criteria are: (1) receiving packed red blood within 4 h of arrival; (2) emergency department (ED) discharge to operating room within 90 min; (3) ED discharge to interventional radiology; (4) ED discharge to intensive care unit (ICU) with ICU length of stay ≥ 3 calendar days; (5) nonprocedural mechanical ventilation within 72 h of arrival; and (6) mortality within 60 h of arrival [75]. NFTI seems a better indicator of major trauma. Thus, the ability of SI to identify severe trauma could be also assessed considering this definition. Current literature is lacking about this.

The strength of our study is the high number of patients considered in the analysis. A previous systematic review has been conducted to investigate the role of SI to predict MT in trauma patients in 2014 [2]. Since that, other 26 studies have been considered. However, we are also aware of some limitations. Retrospective studies have been included in the analysis (only 1 prospective study) and this may be responsible for bias. Most of the included studies considered the classic definition of MT (> 10 RBC units/24 h). This is currently less and less frequent [53] and actually a sort of failure of an integrated hemorrhage control pathway. However, correlating SI with the quality of the hemorrhage control pathway is difficult, and the literature still widely considers MT as evidence of severe hemorrhagic shock. The definition of MT, however, is currently moving from the concept of the number of RBC transfused over a defined time interval to the time-critical character of transfusion management in bleeding trauma patients, which is not limited to RBC but considers early transfusion of any kind of blood product (fibrinogen, plasma, and platelets) [10].

Furthermore, considering the whole adult population, we faced different trauma mechanisms. Statistically, young people are more involved in penetrating traumas than the elderly. Some studies excluded patients with traumatic brain injuries as clinical parameters may be altered independently from hemorrhagic shock while other studies also included this subpopulation. It should also be specified that the elderly population may frequently present other comorbidities that can influence the outcome. For this reason, several authors propose adapting SI to age [35]. All these aspects may contribute to increasing heterogeneity and results should be interpreted with caution.

The records of all the studies considered do not contain information regarding any therapies made by the patient: beta-blockers and anti-hypertensive drugs, analgesics, drugs for the treatment of anxiety, and others that can affect the HR response to hemorrhage and alter other vital signs.

Regarding mortality, the main limitation of our study is represented by the heterogeneity of the different studies in reporting this outcome. There is no uniformity as regards the time in which the patient's mortality was recorded (hospital mortality, at 24 h, 48 h, or 28 days).

## Conclusions

Our study demonstrates that SI may have a weak role to detect the risk for MT in adult trauma patients. On the other hand, regarding mortality, SI may be useful only to identify patients with a low risk of mortality, as for its low sensitivity and high specificity. However, the low/very low quality of evidence due to the retrospective design of the included studies and some sources of heterogeneity does not allow to recommend SI as the sole parameter to consider in predicting MT and mortality in trauma patients. The role of SI adjusted for age for special populations (pediatric and elderly) needs to be further assessed.

## Supplementary Information

**Additional file 1.** Study protocol, supplemental tables and figures.

## Data Availability

The datasets used and/or analysed during the current study are available from the corresponding author on reasonable request.

## Abbreviations

AgeSI: Age shock index
AUC: Area under the curve
BPAI: Blood pressure-age index
ED: Emergency department
GRADE: Grading of Recommendations Assessment, Development and Evaluation
HR: Heart rate
H-SI: Hospital shock index
ICU: Intensive care unit
ISS: Injury Severity Score
LR+: Positive likelihood ratio
LR−: Negative likelihood ratio
MP: Minpulse
MT: Massive blood transfusion
NFTI: Need For Trauma Intervention
PH-SI: Pre-hospital shock index
PMI: Pulse max index
PRISMA-DTA: Preferred Reporting Items for Systematic Reviews and Meta-analyses of diagnostic test accuracy studies
PRISMA-P: Preferred Reporting Items for Systematic Review and Meta-Analysis Protocols
QUADAS-2: Quality assessment of diagnostic accuracy studies
RBC: Red blood cells
RISC II: Revised injury severity classification II
RTS: Revised Trauma Score
SBP: Systolic blood pressure
SI: Shock index
SIPA: Shock index pediatric adjusted
SROC: Summary receiver operating characteristic
TBI: Traumatic brain injury
TRISS: Trauma and injury severity score
