# Supplementary material for: Shock index as predictor of massive transfusion and mortality in patients with trauma: a systematic review and meta-analysis
Source: Crit Care. 2023 Mar 5;27:85. doi: 10.1186/s13054-023-04386-w (PMC9985849; doi:10.1186/s13054-023-04386-w)

SUPPLEMENTAL MATERIAL

Shock index as predictor of massive transfusion and mortality in patients with trauma: a systematic review and meta-analysis

Andrea Carsetti[1,2], Riccardo Antolini[1], Erika Casarotta[1], Elisa Damiani[1,2], Francesco Gasparri[2], Benedetto Marini[2], Erica Adrario[1,2], Abele Donati[1,2]*

[1]Department of Biomedical Sciences and Public Health, Università Politecnica delle Marche, Ancona, Italy
[2]Anesthesia and Intensive Care Unit, Azienda Ospedaliero Universitaria delle Marche, Ancona, Italy

Correspondence: Prof. Andrea Carsetti, e-mail: a.carsetti@univpm.

Table of contents

STUDY PROTOCOL **2**
SEARCH STRATEGY **6**
SUPPLEMENTAL TABLES **7**
SUPPLEMENTAL FIGURES **19**

STUDY PROTOCOL

<u>Study title</u>: Shock Index as a predictor of massive transfusion in patients with trauma: a systematic review and meta-analysis.

<u>Date and version:</u> 26/07/2021 v. 1.0

<u>Sponsor:</u> Università Politecnica delle Marche – Ancona, Italy

<u>Founding:</u> none

<u>Principal investigator:</u>
Andrea Carsetti, MD
Department of Biomedical Sciences and Public Health
Università Politecnica delle Marche – Ancona, Italy
SOD Clinica di Anestesia e Rianimazione Generale, Respiratoria e del Trauma Maggiore
Azienda Ospedaliero Universitaria Ospedali Riuniti di Ancona – Ancona, Italy

Background

Major trauma is an important cause of death, especially in the young population. Hemorrhagic shock is the principal consequence of trauma leading to death. In the early phase of hemorrhagic shock, the organism can compensate for the blood volume loss with vasoconstriction and tachycardia. The early recognition of this condition is fundamental to trigger rapid assessment and treatment to stop hemorrhage and to trigger an appropriate transfusion therapy to rapidly replace blood loss and limit tissue hypoperfusion. Shock Index (SI) is calculated as the ratio between heart rate and systolic blood pressure and several studies showed that it is related to the severity of shock [1]. A higher value of SI was associated with the need for massive transfusion and was related to mortality [1–4]. The current European guidelines on the management of major bleeding and coagulopathy following trauma suggest using SI to assess the severity of hypovolemic shock [5].
Thus, this systematic review aims to synthesize the overall evidence about the capability of SI to predict the need for massive transfusion in patients with trauma, giving stronger evidence for its routine application in clinical practice.

<u>Review questions and hypothesis</u>

The primary research question is: can SI predict the need for massive transfusion in patients with trauma?
We hypothesized that SI is significantly higher in trauma patients that need massive transfusion than in patients that do not need a massive transfusion.
The secondary research question is: can SI predict the mortality of patients with trauma?
We hypothesized that SI is significantly higher in trauma patients who do not survive after trauma than in patients that survive.

Methods

<u>Eligibility criteria for studies</u>

Inclusion criteria:

- Studies enrolling patients with trauma
- Studies reporting SI calculated in the pre-hospital setting or the Emergency Department

Exclusion criteria:

- Studies enrolling population <14 years old

Patient population

Patients >14 years old with trauma.

Primary outcome

The primary outcome of the study is to assess the accuracy of SI to detect the need for massive transfusion in patients with trauma.

Secondary outcome

The secondary outcome is to assess the accuracy of SI to detect mortality in patients with trauma.

Searches strategy

MEDLINE, Scopus, and Web of Science databases will be searched electronically.

The following terms will be included:

1. Shock index
2. Trauma
3. Polytrauma
4. Multiple injur*
5. 2 OR 3 OR 4
6. Hemorrhagic shock
7. Bleeding
8. Hypovolemic shock
9. Hypovolemia
10. 6 OR 7 OR 8 OR 9
11. Transfusion
12. Mortality
13. 1 AND 5 AND 10 AND 11
14. 1 AND 5 AND 10 AND 12

Types of study to be included

Randomized controlled trials and observational trials (prospective and retrospective) will be included.

Conference proceedings, abstracts, case reports, and studies not involving humans will be excluded.

No language restriction will be applied.

Selection of studies

Two investigators will independently perform the first screen (title and abstract), and the full-text screen of the studies retrieved by our search. The same investigators will independently extract the data. Discrepancies at any step of the process (first screening, full-text screening, and data extraction) will be resolved by consensus or by the opinion of a third investigator.

Data extraction

The following data will be extracted from each study:

- Study year
- Study design

- Inclusion and exclusion criteria
- Number of patients enrolled
- Definition of massive transfusion
- Time for mortality outcome recording
- Diagnostic accuracy to predict massive transfusion for SI
    - Sensitivity
    - Specificity
    - Positive predicted value
    - Negative predicted value
    - AUC-ROC
    - Cut-off value
- Diagnostic accuracy to predict mortality for SI
    - Sensitivity
    - Specificity
    - Positive predicted value
    - Negative predicted value
    - AUC-ROC
    - Cut-off value

Risk of bias (quality) assessment

As per the Cochrane DTA handbook [6], the QUADAS-2 tool will be used to assess for risk of bias [7]. The tool will be used by both independent reviewers, and any disagreements will be discussed with the senior author, when necessary. The overall strength and quality of the body of evidence will be assessed according to the GRADE guidelines [8,9].

Strategy for data synthesis

The statistical analysis will be performed using diagmeta package in R (R version 4.2.0 (2022-04-22)), and metandi and midas in STATA (StataCorp 2021; Stata Statistical Software: Release 17; StataCorp LLC). The bivariate model proposed by Reitsma et al. will be used to assess the accuracy of SI to predict MT and mortality and for SROC calculation [4]. In case of multiple cut-offs for SI, the model of multiple thresholds proposed by Steinhuser will be used if appropriate [5].
Quantitative SROC analysis will be performed if five or more studies reported data for the primary/secondary outcome. In the presence of an appropriate number of studies, subgroup analysis considering pre-hospital SI (PH-SI) and SI recorded at hospital admission (H-SI) will be performed. Further subgroup analysis will be considered to investigate the potential sources of heterogeneity.

References:

1. Mutschler M, Nienaber U, Münzberg M, Wölfl C, Schoechl H, Paffrath T, et al. The Shock Index revisited - a fast guide to transfusion requirement? A retrospective analysis on 21,853 patients derived from the TraumaRegister DGU®. Crit. Care. 2013;17.
2. Demuro JP, Simmons S, Jax J, Gianelli SM. Application of the shock index to the prediction of need for hemostasis intervention. Am. J. Emerg. Med. Elsevier Inc.; 2013;31:1260–3.
3. Olaussen A, Blackburn T, Mitra B, Fitzgerald M. Review article: shock index for prediction of critical bleeding post-trauma: a systematic review. Emerg. Med. Australas. Blackwell Publishing;

2014;26:223–8.
4. Lai W-H, Wu S-C, Rau C-S, Kuo P-J, Hsu S-Y, Chen Y-C, et al. Systolic Blood Pressure Lower than Heart Rate upon Arrival at and Departure from the Emergency Department Indicates a Poor Outcome for Adult Trauma Patients. Int. J. Environ. Res. Public Health. MDPI AG; 2016;13.
5. Spahn D, Bouillon B, Cerny V, Duranteau J, Filipescu D, Hunt B, et al. The European guideline on management of major bleeding and coagulopathy following trauma: fifth edition. Crit. care. 2019;23:98.
6. Reitsma J, Rutjes A, Whiting P, Vlassov V, Leeflang M, Deeks J. Chapter 9: Assessing methodological quality. In: Deeks J, Bossuyt P, Gatsonis C, editors. Cochrane Handb. Syst. Rev. Diagnostic Test Accuracy [Internet]. 1.0.0. The Cochrane Collaboration; 2009. Available from: http://srdta.cochrane.org/
7. Whiting PF, Rutjes AWS, Westwood ME, Mallett S, Deeks JJ, Reitsma JB, et al. Quadas-2: A revised tool for the quality assessment of diagnostic accuracy studies. Ann. Intern. Med. 2011;155:529–36.
8. Schünemann H, Mustafa R, Brozek J, Steingart K, Leeflang M, Murad M, et al. GRADE guidelines: 21 part 1. Study design, risk of bias, and indirectness in rating the certainty across a body of evidence for test accuracy. J. Clin. Epidemiol. 2020;122:129–41.
9. Schünemann H, Mustafa R, Brozek J, Steingart K, Leeflang M, Murad M, et al. GRADE guidelines: 21 part 2. Test accuracy: inconsistency, imprecision, publication bias, and other domains for rating the certainty of evidence and presenting it in evidence profiles and summary of findings tables. J. Clin. Epidemiol. 2020;122:142–52.

SEARCH STRATEGY

1. Shock index
2. Trauma
3. Polytrauma
4. Multiple injur*
5. 2 OR 3 OR 4
6. Hemorrhagic shock
7. Bleeding
8. Hypovolemic shock
9. Hypovolemia
10. 6 OR 7 OR 8 OR 9
11. Transfusion
12. Mortality
13. 1 AND 5 AND 10 AND 11
14. 1 AND 5 AND 10 AND 12

SUPPLEMENTAL TABLES

Supplemental Table 1 Characteristics of the selected studies.

Study	Design	Sample size	Shock Index	Inclusion criteria	Exclusion criteria
King et al, 1996	Retrospective	1101	H-SI	≥14 yo	Patients who not required trauma system activation.<br>Patients with severe TBI (GCS <8).<br>Patients with incomplete records or documentation
Zarzaur et al, 2008	Retrospective	16077	H-SI	≥18 yo<br><81 yo, blunt trauma.<br>Bpm>10, SBP>30mmHg in ED	Head or spinal injuries.<br>Greater than 24h from the injury.<br>Incomplete records or documentation
Cannon et al, 2009	Retrospective	2445	PH-SI, H-SI	Moderate or severe trauma with or without hemodynamic stability.<br>Patients were direct arrivals to the ED from the field.	Patients transferred from another facility.<br>Patients who not required trauma system activation.
Vandromme et al, 2011	Retrospective	8111	PH-SI	All trauma patients	Patients transferred from another facility, penetrating trauma, phSBP <90 mmHg

Bruijns et al, 2013	Retrospective	69367	H-SI	≥16 yo	Head or spinal injuries.<br>Unknown injuries.<br>Patients that required either prehospital intubation or cardiopulmonary resuscitation.
Pandit et al, 2013	Retrospective	217190	H-SI	≥65 yo	Patients transferred from another facility.<br>Patients with burn injuries.<br>Isolated TBI.<br>Patients with recorded comorbidity of hypertension.<br>Patients with missing data on vital parameters.
Mutschler et al, 2013	Retrospective	21853	H-SI	≥16 yo, primary admission, complete datasets for SBP, HR and GCS<br>Patients were direct arrivals to the ED from the field.	-
Mitra et al, 2014	Retrospective	1419	PH-SI	Patients transported to hospital directly from the scene of	Patients who received less than 1L crystalloid pre

				injury	hospital.<br>Patients with pre hospital time less than 30 minutes.
Ono et al, 2014	Retrospective	722	H-SI	Trauma required emergency surgery	-
Olaussen et al, 2015	Retrospective	5619	PH-SI, H-SI	≥16 yo; Major trauma (ISS>15)	-
Montoya et al, 2015	Retrospective	666	H-SI	≥18 yo, trauma patients admitted to the institution with shock index taken during admission	>50 yo, patients with recorded comorbidity of hypertension.<br>Patients with metabolic syndrome.
Yang et al, 2016	Prospective	677	PH-SI	≥18 yo, SI≥0.62 in the field	-
Pottecher et al, 2016	Retrospective	2557	PH-SI	Major trauma	Intractable cardiac arrest in the field, non-emergency medical services prehospital transport, unknown prehospital transport
Frohlich et al, 2016	Retrospective	38162	H-SI	≥14 yo, primary admission, admission to an intensive care unit (ICU) and complete datasets for systolic blood pressure,	-

				heart rate and GCS	
Rau et al, 2016	Retrospective	2490	H-SI	Patients who had received transfusion of packed red blood cells or whole blood at the ED within 24h	Patients with incomplete registered data were excluded
Kim et al, 2016	Retrospective	45880	H-SI	≥65 yo	Patients dead upon arrival at the ED, isolated TBI. Patients with non-traumatic injuries (burn, drowning or drug intoxication)
David et al, 2017	Retrospective	485	PH-SI	All patients having a thromboelastomeric evaluation and/or who received a blood product or coagulation factors concentrates	Patients treated with anticoagulant agents. Patients without standard laboratory test for haemostasis.
Schroll et al, 2018	Retrospective	644	H-SI	≥18 yo	Traumatic brain injury
El Menyar et al, 2018	Retrospective	8710	H-SI	trauma patients who received blood transfusion in the emergency room	<14yo, patients with incomplete or irrelevant clinical data
Wu et al, 2018	Retrospective	18750	H-SI	≥20 yo who sustained a traumatic injury	Patients with burn injuries, incomplete registered data
Bhandarkar et al, 2018	Retrospective	8886	H-SI	All patients presenting to the	Patients who were dead on arrival

				casualty department with history of injury with a mechanism of road traffic, railway, fall, assault, or burns.	
Figueiredo et al, 2018	Retrospective	6402	PH-SI, H-SI	≥18 yo. Trauma patients directly admitted from the trauma scene if they had at least one prehospital Hb measurement	Patients with incomplete data
Zhu et al, 2019	Retrospective	157	PH-SI	≥18 yo, <89 yo and activation of massive transfusion protocol after meeting criteria	Prisoners; Pregnant women; nontraumatic hemorrhage.
El-Menyar et al, 2019	Retrospective	572	H-SI	All adult patients who were transported directly to ED from the scene. Abdominal trauma patients.	Patients transferred from another facility. Patients with prehospital cardiac arrest. Patients with incomplete data
Jouini et al, 2019	Prospective	290	H-SI	≥18 yo, trauma grave.	Beta-blocker users. Pregnant women. Patients dead at the admission
Jehan et al, 2019	Retrospective	144951	PH-SI	≥18 yo	Patients transferred from another

					facility
Wang et al, 2020	Retrospective	1007	PH-SI	≥18 yo with blunt or penetrating injuries	Patients transferred from another facility.<br>Patients with pre hospital cardiac arrest.<br>Patients without blunt or penetrating injury mechanism.
Marenco et al, 2020	Retrospective	4008	H-SI	Adult combat trauma causalities	Patients without initial arrival vital signs recorded.<br>Patients whose first set of complete vital signs was recorded at a tertiary care center.
Kheirbek et al, 2021	Retrospective	544	PH-SI	Adult patients who were evaluated for full trauma team activation	Patients transferred from another facility, patients with missing values
Kim et al, 2021	Retrospective	21534	PH-SI	≥18 yo, <85 yo.<br>Abdominal trauma.<br>Trauma to upper and lower limbs.	Patients from China, Indonesia, Japan, Philippines, Thailand and Vietnam.<br>Prehospital cardiac arrest.<br>Patients transferred from another

					facility,<br>Patients with anatomical injury in the head, face, neck and spine. Patients with incomplete data and unknown outcome.
Chowdhury et al, 2021	Retrospective	6667	H-SI	All trauma patients with principal diagnosis of injury and one of the following: death in the ED, inpatient admission, inpatient death following injury, admission to the intensive care unit.	Superficial injury and/or amputation of single finger.<br>Length of stay <3 days apart from death or admission to the ICU.<br>Burns < 10% total body surface area.<br>Injury date more than 3 days before admission to the first hospital.
Asim et al, 2022	Retrospective	1645	H-SI	All adult (aged >18yo) blunt thoracic trauma patients with chest AIS <1 admitted to the hospital.	Prehospital cardiac arrest, missing initial vital signs, pediatric cases, penetrating trauma, patients undergoing orthopedic surgery.
Kakimoto et al, 2022	Retrospective	9753	H-SI	Trauma patients.<br>>18 yo,<br>Patients visited the emergency	Cardiac arrest on hospital arrival.<br>Patients with burn injuries.

				department on foot or using a private car	Unknown survival outcomes.
Turan et al, 2022	Retrospective	102	H-SI	>18 yo.<br>Blunt thoracoabdominal trauma.	Patients with missing data, transferred from another institution, who underwent cardiopulmonary resuscitation before coming to ED.<br>Patients with diabetes mellitus or renal failure.<br>Patients taking antihypertensive or potassium-modifying medication

SI: Shock Index; PH-SI: Pre-Hospital Shock Index; H-SI: Hospital-Shock Index; yo: years old; TBI: traumatic brain injury; ICU: intensive care unit.

Supplemental Table 2 Outcome definition of selected studies.

Study	Sample size	Outcome	Definition of MT	Definition of mortality
King et al, 1996	1101	Mortality		24h mortality
Zarzaur et al, 2008	16077	Mortality		48h mortality
Cannon et al, 2009	1166	Mortality		?
Vandromme et al, 2011	8111	MT, mortality	≥10 RBC units within 24h	In-hospital mortality
Bruijns et al, 2013	69367	Mortality		48h mortality
Pandit et al, 2013	217190	Mortality		In-hospital mortality
Mutschler et al, 2013	21853	Mortality		Early mortality
Mitra et al, 2014	1419	MT	≥5 RBC units within 4h	
Ono et al, 2014	722	Mortality		?
Olaussen et al, 2015	5619	MT	≥5 RBC units within 4h	
Montoya et al, 2015	666	Mortality		24h mortality
Yang et al, 2016	677	Mortality		?
Pottecher et al, 2016	2557	MT	≥10 RBC units within 24h	
Frohlich et al, 2016	38162	Mortality		In-hospital mortality
Rau et al, 2016	2490	MT	≥10 RBC units within 24h	
Kim et al, 2016	45880	Mortality		In-hospital mortality
David et al, 2017	485	MT	≥10 RBC units within 24h	
Schroll et al, 2018	644	MT	≥10 RBC units within 24h	
El Menyar et al, 2018	8710	MT, mortality	≥10 RBC units within 24h	In-hospital mortality
Wu et al, 2018	18750	Mortality		In-hospital mortality
Bhandarkar et al, 2018	8886	Mortality		?
Figueiredo et al, 2018	6402	MT	≥4 RBC units within 6h	

Zhu et al, 2019	157	MT	≥10 RBC units within 24h	
El-Menyar et al, 2019	572	MT, mortality	Replacement of the patient’s total blood volume (approximately 5 L) over 24h. Administration of >40ml/kg PRBC in 2h	In-hospital mortality
Jouini et al, 2019	290	Mortality		30 days mortality
Jehan et al, 2019	144951	MT, mortality	≥10 RBC units within 24h	ED mortality, In-hospital mortality
Wang et al, 2020	1007	MT, Mortality	≥10 RBC units within 24h	24h mortality
Marenco et al, 2020	4008	MT, mortality	≥10 RBC units within 24h	?
Kheirbek et al, 2021	544	Mortality		ED mortality, 24h mortality
Kim et al, 2021	21534	MT, mortality	>4000 ml in 24h	In-hospital mortality
Chowdhury et al, 2021	6667	Mortality		In-hospital mortality
Asim et al, 2022	1645	Mortality		In-hospital mortality
Kakimoto et al, 2022	9753	Mortality		In-hospital mortality
Turan et al, 2022	102	Mortality		?

?: not clearly define; MT: massive transfusion; ED: emergency department; RBC: Red blood cell.

Supplemental Table 3 Quality of evidence for Shock Index to predict massive transfusion in adult patients with trauma.

Question: Should Shock Index be used to diagnose need for massive transfusion in adult patients with trauma?

Sensitivity	0.68 (95% CI: 0.57 to 0.76)
Specificity	0.84 (95% CI: 0.79 to 0.88)

Prevalences	2%	3%	5%

Outcome	№ of studies (№ of patients)	Study design	Factors that may decrease certainty of evidence					Effect per 1.000 patients tested			Test accuracy CoE
			Risk of bias	Indirectness	Inconsistency	Imprecision	Publication bias	pre-test probability of 2%	pre-test probability of 3%	pre-test probability of 5%	
**True positives** (patients with need for massive transfusion)	17 studies 6775 patients	case-control type accuracy study	serious[a]	serious[b]	not serious	not serious	none	14 (11 to 15)	20 (17 to 23)	34 (28 to 38)	⨁⨁◯◯ Low
**False negatives** (patients incorrectly classified as not having need for massive transfusion)								6 (5 to 9)	10 (7 to 13)	16 (12 to 22)	
**True negatives** (patients without need for massive transfusion)	17 studies 213912 patients	case-control type accuracy study	serious[a]	serious[b]	not serious	not serious	none	823 (774 to 862)	815 (766 to 854)	798 (751 to 836)	⨁⨁◯◯ Low
**False positives** (patients incorrectly classified as having need for massive transfusion)								157 (118 to 206)	155 (116 to 204)	152 (114 to 199)	

Explanations

a. Retrospective studies included

b. Heterogeneity in patients characteristics

Supplemental Table 4 Quality of evidence for Shock Index to predict mortality in adult patients with trauma.

Question: Should Shock Index be used to diagnose mortality risk in adult patients with trauma?

Sensitivity	0.36 (95% CI: 0.24 to 0.50)
Specificity	0.74 (95% CI: 0.66 to 0.81)

Prevalences	10%	15%	20%

Outcome	№ of studies (№ of patients)	Study design	Factors that may decrease certainty of evidence					Effect per 1.000 patients tested			Test accuracy CoE
			Risk of bias	Indirectness	Inconsistency	Imprecision	Publication bias	pre-test probability of 10%	pre-test probability of 15%	pre-test probability of 20%	
**True positives** (patients with mortality risk)	26 studies 91844 patients	case-control type accuracy study	serious[a]	serious[b]	not serious	serious[c]	none	36 (24 to 50)	54 (36 to 75)	72 (48 to 100)	⨁◯◯◯ Very low
**False negatives** (patients incorrectly classified as not having mortality risk)								64 (50 to 76)	96 (75 to 114)	128 (100 to 152)	
**True negatives** (patients without mortality risk)	26 studies 557826 patients	case-control type accuracy study	serious[a]	serious[b]	not serious	serious[c]	none	668 (590 to 732)	631 (558 to 691)	594 (525 to 650)	⨁◯◯◯ Very low
**False positives** (patients incorrectly classified as having mortality risk)								232 (168 to 310)	219 (159 to 292)	206 (150 to 275)	

Explanations

a. Retrospective studies included

b. Heterogeneity in patients characteristics

c. Heterogeneity in outcome definition

SUPPLEMENTAL FIGURES

Supplemental Figure 1 Forest plot for pre-hospital SI showing the individual study sensitivity and specificity (A) and SROC curve for massive transfusion (B).

A.

Study	TP	FP	FN	TN	Sensitivity (95% CI)	Specificity (95% CI)
David et al 2017	21	134	1	329	0.95 [0.77, 1.00]	0.71 [0.67, 0.75]
Figueiredo et al 2018	468	960	287	4687	0.62 [0.58, 0.65]	0.83 [0.82, 0.84]
Jehan et al 2019 PH-SI	3297	9893	158	131603	0.95 [0.95, 0.96]	0.93 [0.93, 0.93]
Kim et al 2021	22	1630	36	19846	0.38 [0.26, 0.52]	0.92 [0.92, 0.93]
Mitra et al 2014	90	117	98	1114	0.48 [0.41, 0.55]	0.90 [0.89, 0.92]
Olaussen et al 2015	186	900	136	4397	0.58 [0.52, 0.63]	0.83 [0.82, 0.84]
Pottecher et al 2016	38	350	18	2151	0.68 [0.54, 0.80]	0.86 [0.85, 0.87]
Vandromme et al 2011 cut-off 0.5	249	7160	27	675	0.90 [0.86, 0.93]	0.09 [0.08, 0.09]
Vandromme et al 2011 cut-off 0.7	171	4158	105	3677	0.62 [0.56, 0.68]	0.47 [0.46, 0.48]
Vandromme et al 2011 cut-off 0.9	95	1396	181	6439	0.34 [0.29, 0.40]	0.82 [0.81, 0.83]
Vandromme et al 2011 cut-off 1.1	47	269	229	7566	0.17 [0.13, 0.22]	0.97 [0.96, 0.97]
Vandromme et al 2011 cut-off 1.3	12	56	264	7779	0.04 [0.02, 0.07]	0.99 [0.99, 0.99]
Wang et al 2020	51	214	27	715	0.65 [0.54, 0.76]	0.77 [0.74, 0.80]
Zhu et al 2019	68	31	34	24	0.67 [0.57, 0.76]	0.44 [0.30, 0.58]

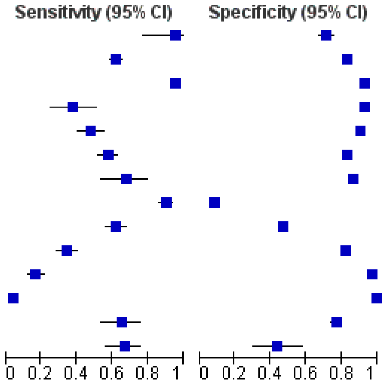

B.

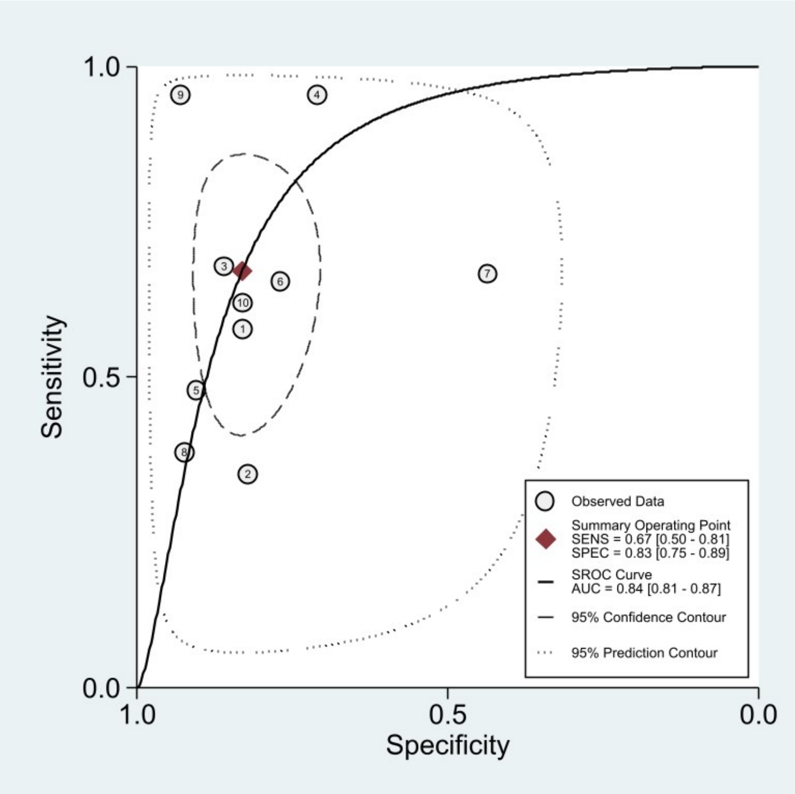

Supplemental Figure 2 Forest plot for hospital SI showing the individual study sensitivity and specificity (A) and SROC curve for massive transfusion (B).

A.

Study	TP	FP	FN	TN	Sensitivity (95% CI)	Specificity (95% CI)
El Menyar et al 2018	71	1464	18	7157	0.80 [0.70, 0.88]	0.83 [0.82, 0.84]
El Menyar et al 2019 cut-off 0.8	63	226	11	272	0.85 [0.75, 0.92]	0.55 [0.50, 0.59]
El Menyar et al 2019 cut-off 0.9	56	147	18	351	0.76 [0.64, 0.85]	0.70 [0.66, 0.74]
El Menyar et al 2019 cut-off 1	47	104	27	394	0.64 [0.52, 0.74]	0.79 [0.75, 0.83]
Figueiredo et al 2018	468	791	287	4856	0.62 [0.58, 0.65]	0.86 [0.85, 0.87]
Marenco et al 2020 cut-off 0.8	79	859	11	3059	0.88 [0.79, 0.94]	0.78 [0.77, 0.79]
Marenco et al 2020 cut-off 0.9	72	482	18	3436	0.80 [0.70, 0.88]	0.88 [0.87, 0.89]
Marenco et al 2020 cut-off 1	72	482	18	3436	0.80 [0.70, 0.88]	0.88 [0.87, 0.89]
Olaussen et al 2015	168	367	154	4930	0.52 [0.47, 0.58]	0.93 [0.92, 0.94]
Rau et al 2016	56	296	43	2095	0.57 [0.46, 0.67]	0.88 [0.86, 0.89]
Schroll et al 2018	23	114	11	496	0.68 [0.49, 0.83]	0.81 [0.78, 0.84]

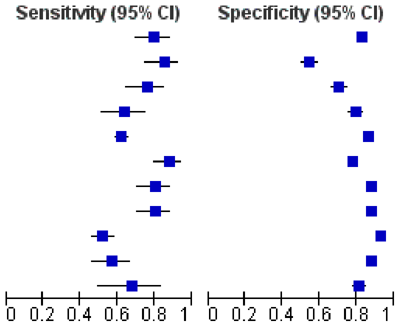

B.

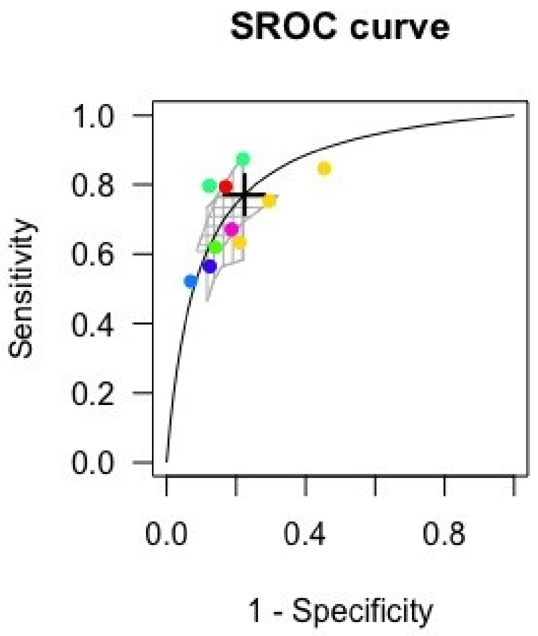

Supplemental Figure 3 Forest plot for pre-hospital SI showing the individual study sensitivity and specificity (A) and SROC curve for mortality (B).

A.

Study	TP	FP	FN	TN	Sensitivity (95% CI)	Specificity (95% CI)
Cannon et al 2009	35	357	45	729	0.44 [0.33, 0.55]	0.67 [0.64, 0.70]
Jehan et al 2019 PH-SI	396	12794	1054	130707	0.27 [0.25, 0.30]	0.91 [0.91, 0.91]
Kheirberk et al 2021	23	74	81	366	0.22 [0.15, 0.31]	0.83 [0.79, 0.87]
Kim et al 2021	87	1565	126	19756	0.41 [0.34, 0.48]	0.93 [0.92, 0.93]
Vandromme et al 2011 cut-off 0.5	159	7250	28	674	0.85 [0.79, 0.90]	0.09 [0.08, 0.09]
Vandromme et al 2011 cut-off 0.7	100	4229	87	3695	0.53 [0.46, 0.61]	0.47 [0.46, 0.48]
Vandromme et al 2011 cut-off 0.9	22	294	165	7630	0.12 [0.08, 0.17]	0.96 [0.96, 0.97]
Vandromme et al 2011 cut-off 1.1	165	151	22	7773	0.88 [0.83, 0.92]	0.98 [0.98, 0.98]
Vandromme et al 2011 cut-off 1.3	7	61	180	7863	0.04 [0.02, 0.08]	0.99 [0.99, 0.99]
Wang et al 2020	8	68	22	909	0.27 [0.12, 0.46]	0.93 [0.91, 0.95]
Yang et al 2016	10	273	2	392	0.83 [0.52, 0.98]	0.59 [0.55, 0.63]

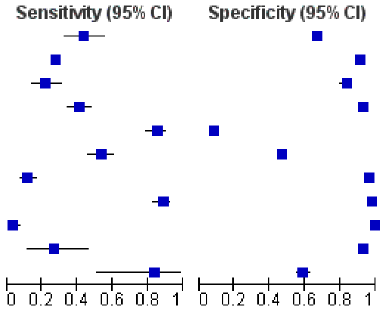

B.

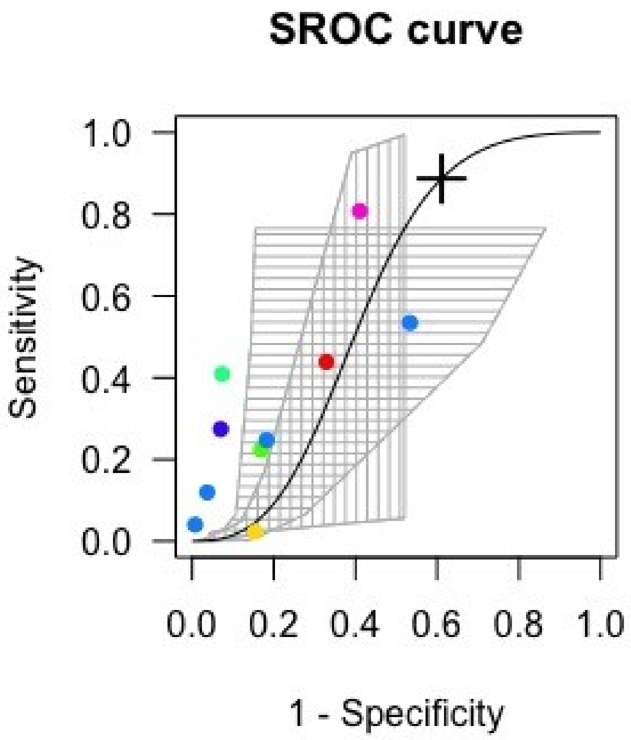

Supplemental Figure 4 Forest plot for hospital SI showing the individual study sensitivity and specificity (A) and SROC curve for mortality (B).

A.

Study	TP	FP	FN	TN	Sensitivity (95% CI)	Specificity (95% CI)
Asim M et al 2022	33	370	10	1232	0.77 [0.61, 0.88]	0.77 [0.75, 0.79]
Bhandarkar et al 2018 cut-off 0.6	1820	6212	207	647	0.90 [0.88, 0.91]	0.09 [0.09, 0.10]
Bhandarkar et al 2018 cut-off 1	557	863	1470	5996	0.27 [0.26, 0.29]	0.87 [0.87, 0.88]
Bhandarkar et al 2018 cut-off 1.4	157	155	1870	6704	0.08 [0.07, 0.09]	0.98 [0.97, 0.98]
Bruijns et al 2013 cut-off 0.8	120	6910	145	62192	0.45 [0.39, 0.51]	0.90 [0.90, 0.90]
Bruijns et al 2013 cut-off 0.9	99	3455	166	65647	0.37 [0.32, 0.43]	0.95 [0.95, 0.95]
Cannon et al 2009	84	444	121	1796	0.41 [0.34, 0.48]	0.80 [0.78, 0.82]
Chowdhury et al 2021	76	832	161	5598	0.32 [0.26, 0.38]	0.87 [0.86, 0.88]
El Menyar et al 2018	109	1426	222	6953	0.33 [0.28, 0.38]	0.83 [0.82, 0.84]
El Menyar et al 2019 cut-off 0.8	41	248	9	274	0.82 [0.69, 0.91]	0.52 [0.48, 0.57]
El Menyar et al 2019 cut-off 0.9	35	168	15	354	0.70 [0.55, 0.82]	0.68 [0.64, 0.72]
El Menyar et al 2019 cut-off 1	31	120	19	402	0.62 [0.47, 0.75]	0.77 [0.73, 0.81]
Frohlich et al 2016 cut-off 0.6	3366	22670	1211	10915	0.74 [0.72, 0.75]	0.32 [0.32, 0.33]
Frohlich et al 2016 cut-off 1	1470	3925	3107	29660	0.32 [0.31, 0.33]	0.88 [0.88, 0.89]
Frohlich et al 2016 cut-off 1.4	629	1010	3948	32575	0.14 [0.13, 0.15]	0.97 [0.97, 0.97]
Jouini et al 2019	28	31	35	196	0.44 [0.32, 0.58]	0.86 [0.81, 0.91]
Kakimoto K. et al 2022	17	466	113	9157	0.13 [0.08, 0.20]	0.95 [0.95, 0.96]
Kim et al 2016 cut-off 0.8	312	4807	221	40540	0.59 [0.54, 0.63]	0.89 [0.89, 0.90]
Kim et al 2016 cut-off 0.9	261	2086	272	43261	0.49 [0.45, 0.53]	0.95 [0.95, 0.96]
Kim et al 2016 cut-off 1	212	1043	321	44304	0.40 [0.36, 0.44]	0.98 [0.98, 0.98]
King et al 1996	4	66	3	1028	0.57 [0.18, 0.90]	0.94 [0.92, 0.95]
Marenco et al 2020 cut-off 0.8	43	895	20	3050	0.68 [0.55, 0.79]	0.77 [0.76, 0.79]
Montoya et al 2015	66	45	17	538	0.80 [0.69, 0.88]	0.92 [0.90, 0.94]
Mutschler et al 2013 cut-off 0.6	2106	13265	712	5770	0.75 [0.73, 0.76]	0.30 [0.30, 0.31]
Mutschler et al 2013 cut-off 1	927	2347	1891	16688	0.33 [0.31, 0.35]	0.88 [0.87, 0.88]
Mutschler et al 2013 cut-off 1.4	402	600	2416	18435	0.14 [0.13, 0.16]	0.97 [0.97, 0.97]
Ono et al 2014	24	133	39	526	0.38 [0.26, 0.51]	0.80 [0.77, 0.83]
Pandit et al 2013	1133	5452	7819	202786	0.13 [0.12, 0.13]	0.97 [0.97, 0.97]
Turan et al 2022	9	21	2	70	0.82 [0.48, 0.98]	0.77 [0.67, 0.85]
Wu et al 2018	443	13047	59	5201	0.88 [0.85, 0.91]	0.29 [0.28, 0.29]
Zarzaur et al 2008	149	3333	55	12540	0.73 [0.66, 0.79]	0.79 [0.78, 0.80]

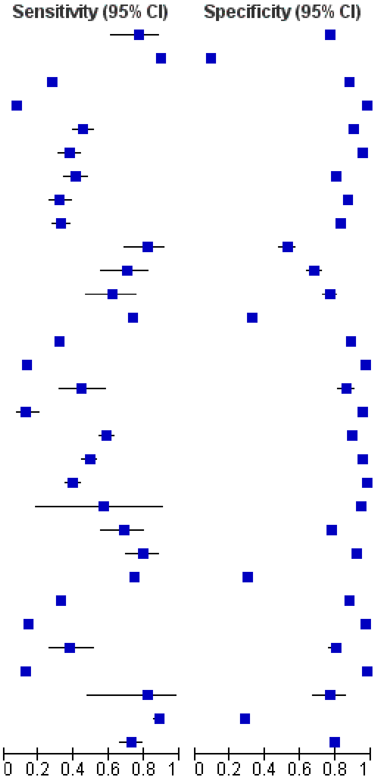

B.

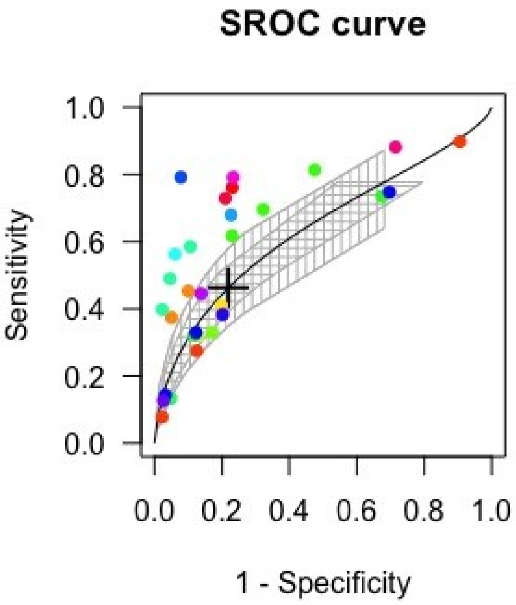

Supplemental Figure 5 Forest plot for SI showing the individual study sensitivity and specificity (A) and SROC curve for in-hospital mortality (B).

A.

Study	TP	FP	FN	TN	Sensitivity (95% CI)	Specificity (95% CI)
Asim M et al 2022	33	370	10	1232	0.77 [0.61, 0.88]	0.77 [0.75, 0.79]
Chowdhury et al 2021	76	832	161	5598	0.32 [0.26, 0.38]	0.87 [0.86, 0.88]
El Menyar et al 2018	109	1426	222	6953	0.33 [0.28, 0.38]	0.83 [0.82, 0.84]
El Menyar et al 2019 cut-off 0.8	41	248	9	274	0.82 [0.69, 0.91]	0.52 [0.48, 0.57]
El Menyar et al 2019 cut-off 0.9	35	168	15	354	0.70 [0.55, 0.82]	0.68 [0.64, 0.72]
El Menyar et al 2019 cut-off 1	31	120	19	402	0.62 [0.47, 0.75]	0.77 [0.73, 0.81]
Frohlich et al 2016 cut-off 0.6	3366	22670	1211	10915	0.74 [0.72, 0.75]	0.32 [0.32, 0.33]
Frohlich et al 2016 cut-off 1	1470	3925	3107	29660	0.32 [0.31, 0.33]	0.88 [0.88, 0.89]
Frohlich et al 2016 cut-off 1.4	629	1010	3948	32575	0.14 [0.13, 0.15]	0.97 [0.97, 0.97]
Jehan et al 2019	1622	11568	68512	63249	0.02 [0.02, 0.02]	0.85 [0.84, 0.85]
Kakimoto K. et al 2022	17	466	113	9157	0.13 [0.08, 0.20]	0.95 [0.95, 0.96]
Kim et al 2016 cut-off 0.8	312	4807	221	40540	0.59 [0.54, 0.63]	0.89 [0.89, 0.90]
Kim et al 2016 cut-off 0.9	261	2086	272	43261	0.49 [0.45, 0.53]	0.95 [0.95, 0.96]
Kim et al 2016 cut-off 1	212	1043	321	44304	0.40 [0.36, 0.44]	0.98 [0.98, 0.98]
Kim et al 2021	87	1565	126	19756	0.41 [0.34, 0.48]	0.93 [0.92, 0.93]
Pandit et al 2013	1133	5452	7819	202786	0.13 [0.12, 0.13]	0.97 [0.97, 0.97]
Vandromme et al 2011 cut-off 0.5	159	7250	28	674	0.85 [0.79, 0.90]	0.09 [0.08, 0.09]
Vandromme et al 2011 cut-off 0.7	100	4229	87	3695	0.53 [0.46, 0.61]	0.47 [0.46, 0.48]
Vandromme et al 2011 cut-off 0.9	46	1445	141	6479	0.25 [0.19, 0.31]	0.82 [0.81, 0.83]
Vandromme et al 2011 cut-off 1.1	22	294	165	7630	0.12 [0.08, 0.17]	0.96 [0.96, 0.97]
Vandromme et al 2011 cut-off 1.3	7	61	180	7863	0.04 [0.02, 0.08]	0.99 [0.99, 0.99]
Wu et al 2018	443	13047	59	5201	0.88 [0.85, 0.91]	0.29 [0.28, 0.29]

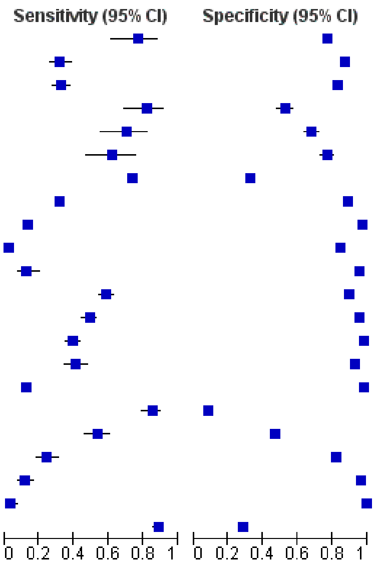

B.

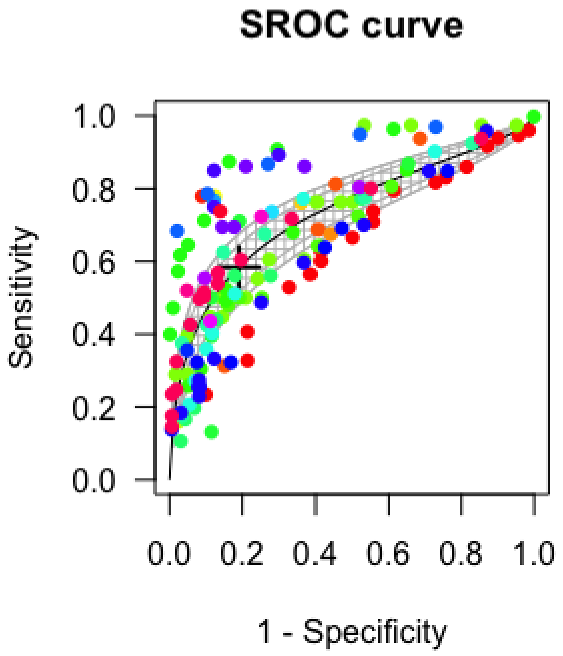

Supplement: Supplementary file 1 — Additional file 1. Study protocol, supplemental tables and figures. [file 13054_2023_4386_MOESM1_ESM.pdf]
